# Blood Meals With Active and Heat-Inactivated Serum Modifies the Gene Expression and Microbiome of *Aedes albopictus*

**DOI:** 10.3389/fmicb.2021.724345

**Published:** 2021-09-09

**Authors:** Arley Calle-Tobón, Andres F. Holguin-Rocha, Celois Moore, Meagan Rippee-Brooks, Paula Rozo-Lopez, Jania Harrod, Soheila Fatehi, Guillermo L. Rua-Uribe, Yoonseong Park, Berlin Londoño-Rentería

**Affiliations:** ^1^Department of Entomology, Kansas State University, Manhattan, KS, United States; ^2^Grupo Entomología Médica, Universidad de Antioquia, Medellín, Colombia; ^3^Department of Biology, Missouri State University, Springfield, MO, United States

**Keywords:** RNA-sequencing, tiger-mosquito, *Aedes*-mosquitoes, RNA viruses, *Wolbachia*, complement-system, immunity

## Abstract

The Asian “tiger mosquito” *Aedes albopictus* is currently the most widely distributed disease-transmitting mosquito in the world. Its geographical expansion has also allowed the expansion of multiple arboviruses like dengue, Zika, and chikungunya, to higher latitudes. Due to the enormous risk to global public health caused by mosquitoes species vectors of human disease, and the challenges in slowing their expansion, it is necessary to develop new and environmentally friendly vector control strategies. Among these, host-associated microbiome-based strategies have emerged as promising options. In this study, we performed an RNA-seq analysis on dissected abdomens of *Ae. albopictus* females from Manhattan, KS, United States fed with sugar and human blood containing either normal or heat-inactivated serum, to evaluate the effect of heat inactivation on gene expression, the bacteriome transcripts and the RNA virome of this mosquito species. Our results showed at least 600 genes with modified expression profile when mosquitoes were fed with normal vs. heat-inactivated-containing blood. These genes were mainly involved in immunity, oxidative stress, lipid metabolism, and oogenesis. Also, we observed bacteriome changes with an increase in transcripts of Actinobacteria, Rhodospirillaceae, and Anaplasmataceae at 6 h post-feeding. We also found that feeding with normal blood seems to particularly influence *Wolbachia* metabolism, demonstrated by a significant increase in transcripts of this bacteria in mosquitoes fed with blood containing normal serum. However, no differences were observed in the virome core of this mosquito population. These results suggest that heat and further inactivation of complement proteins in human serum may have profound effect on mosquito and microbiome metabolism, which could influence interpretation of the pathogen-host interaction findings when using this type of reagents specially when measuring the effect of *Wolbachia* in vector competence.

## Introduction

Currently, *Aedes* (*Stegomyia*) *albopictus* (Skuse, 1894) is considered the medically important most invasive mosquito species in the world (Benedict et al., 2007). In the last 30 years, it has rapidly spread from its native Asian continent across the world and it can be found in countries on every continent except Antarctica (Enserink, 2008; Ding et al., 2018). The expansion from its traditional habitat of tropical and sub-tropical regions to much cooler temperate regions has been attributed to its ecological plasticity, its ability to enter diapause to escape unfavorable seasonal conditions (Kraemer et al., 2019). These factors have further increased the risk of outbreaks in areas where mosquito-borne viral diseases are uncommon, such as Northern America and temperate Europe (Moreno-Madriñán and Turell, 2017, 2018; Barzon, 2018; Adams et al., 2019; Robert et al., 2020). In the United States, *Ae. albopictus* was first registered in 1987 and its presence has been reported in at least 40 States (Moore and Mitchell, 1997; Hahn et al., 2017). Needless to say that *Ae. albopictus* has also been responsible for several outbreaks of dengue (DENV) and chikungunya virus (CHIKV) in tropical areas around the globe (Rezza, 2012; Luo et al., 2017; Lindh et al., 2019).

This mosquito has an opportunistic feeding pattern (Delatte et al., 2010; Kamgang et al., 2012), which increases the risk of transmission of zoonotic diseases to humans. Due to the lack of therapeutic options or widely available vaccines against human arbovirus infections, control of mosquito population is the most used way to prevent diseases transmission (Mahmud et al., 2019). To halter expansion of this mosquito and control pathogen transmitted by it, we need a better understanding of its biology that will lead to innovative and effective control strategies. Pathogens transmission to and from the vertebrate occurs mainly through the bite of female mosquitoes while obtaining their blood meal required to produce eggs. This blood meal is then digested in the midgut to provide the nutrients, but may also increase oxidative stress and cell damage by human active components included in it (Pakpour et al., 2014). To prevent significant damage, mosquitoes adopt different strategies (Khattab et al., 2015) and such active blood derived factors are often, neutralized by enzymes and other molecules in the midgut (Khattab et al., 2015). More importantly, the midgut represents the first barrier against infection with pathogens, so that midgut epithelial integrity as well as proper cellular regenerative process play important roles in pathogen permissiveness and vector competence. Specifically, a delay in the proliferative repair system to preserve the midgut barrier may increase susceptibility to infection (Taracena et al., 2018; Hixson et al., 2021).

Also, because blood meal factors, can remain active for several hours interacting with mosquito cells, human pathogens or even its microbiome mosquito physiology may be affected (Margos et al., 2001; Pakpour et al., 2014). For instance, active complement in midgut decreases pathogen populations present in the blood meal, representing one of the main factors of reduction in *Plasmodium* population in *Anopheles* mosquitoes (Smith et al., 2014), and previous studies also suggest that the presence of active complement factors in a blood meal modifies gene expression of multiple genes related to the immune system in *Aedes aegypti* mosquitoes (Giraldo-Calderón et al., 2020). However, the latest study did not explore the effect of active complement on the microbial communities present in mosquitoes. Blood meal acquisition induces drastic modifications in the mosquito bacterial communities that can be observed from 24 h after feeding (Hegde et al., 2018). Although the microbiome of mosquitoes can be highly variable, both within and between species, it is often dominated by relatively few genera. For mosquitoes, microbiota-host interactions can influence a diverse number of physiological traits including immunity, reproduction, survival, and vector competence (Scolari et al., 2019; Seabournid et al., 2020). Due to this effect, the interest in mosquitoes microbiome manipulation to control the transmission of vector borne diseases has increased in recent years. For example, some endosymbionts Alphaproteobacteria *Wolbachia* sp. strains, when introduced into non-natural hosts, show the ability to decrease susceptibility of *Aedes* sp. and *Anopheles* sp. mosquitoes to arboviruses and *Plasmodium* sp., respectively (Ippolito et al., 2018; Kalappa et al., 2018; O’Neill, 2018). The exact mechanism that allows *Wolbachia* to decrease vector competence is still unclear. However, factors such as a rise in host immune response, elevation in host methyltransferase to reduce viral production and suppression of key lipids required for viral replication have been identified as potentially important mechanisms (Caragata et al., 2016; Bhattacharya et al., 2017; Geoghegan et al., 2017).

To date, most mosquito microbiota studies have been focused on the bacterial composition and their role in mosquitoes biology. However, numerous insect-specific viruses (ISV) have been reported in culicids with increasing evidence suggesting that ISVs influence mosquito physiology as well as its ability to transmit important arboviruses (Öhlund et al., 2019). In this regard, virome studies in *Ae. albopictus* virome revealed that this species harbors viruses from multiple families such as *Flaviviridae*, *Totiviridae*, *Iflaviridae*, *Virgaviridae*, *Rhabdoviridae*, and *Nodaviridae*, that remain stable in mosquito populations, apparently transmitted vertically (Zakrzewski et al., 2018; Kubacki et al., 2020; Shi et al., 2020). Hence, investigating whether active blood factors in the mosquito midgut affect mosquito microbiota, virome and their transcriptome may provide new strategies for mosquito and arbovirus control (Gao et al., 2020). The objective of this study was to evaluate whether the transcriptome of *Ae. albopictus* is affected by feeding with normal or heat-inactivated serum-containing blood, and to determine if different type of meals are associated with modifications in the microbial communities within the mosquito. We consider our approach as a part of meta-transcriptomics (Cox et al., 2017), a fast and robust protocol for meta-taxonomic analysis using RNA-seq data, that provide the guideline for further study (Cox et al., 2017). The results generated from this study provide new information on the effect of human blood on this species and should form the basis for future investigations on the biology, microbiome, and vector competence of *Ae. albopictus.*

## Results

### Differential Gene Expression After Blood Meal

**FIGURE 1:**
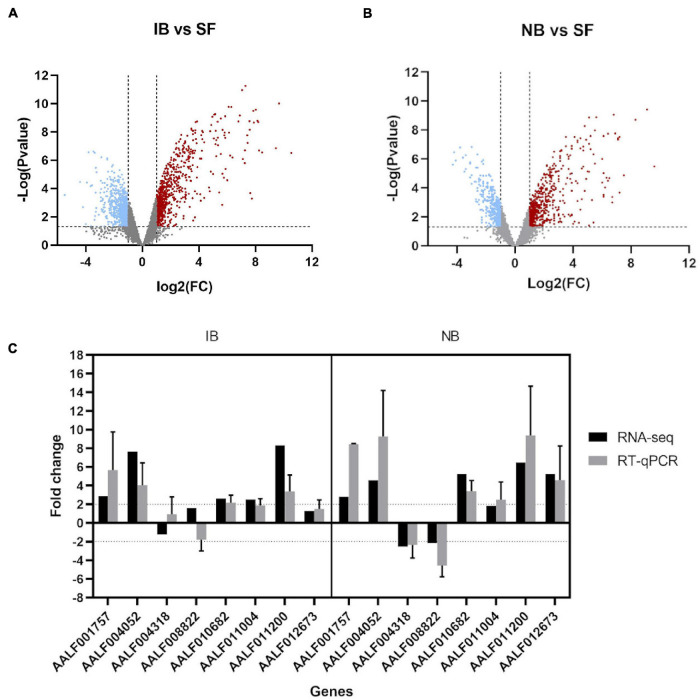
Transcriptome analysis of *Ae. albopictus* fed with inactivated blood and normal blood. **(A)** Volcano plots for the differentially expressed genes (DEGs), Inactivated Blood vs. Sugar Fed. Upregulated (red plots) and downregulated (blue plots) DEGs. **(B)** Volcano plots for the DEGs, Normal Blood vs. Sugar Fed. **(C)** Fold change of eight immune-related genes expression for RNA-seq and qPCR analysis. qPCR data are presented as FC means (±SE) of three technical and three biological replicates.

A total of 12 libraries were generated and sequenced from *Ae. albopictus* abdomens. Mosquitoes were divided according to the meal they received into sugar-fed (SF), inactivated blood-fed (IB), or normal blood-fed (NB). Each feeding treatment was represented by four libraries, and a total of 314,422,593 single-reads were retained after read cleaning with a length average of 137 base pairs (bp). Differential expression analyses showed significant differences in the expression level among treatments (Differentially Expressed Genes or DEG). Specifically, we observed that when comparing IB and SF, a total of 1,527 genes were found differentially expressed (Log2FC > | 1| and *p* ≤ 0.05) (Figure 1A) with 752 upregulated and 775 downregulated. The comparison between NB and SF groups showed a total of 1,038 DEG of which 612 were upregulated and 426 downregulated (Figure 1B). Within the genes observed with differential expression, 447 upregulated and 414 downregulated were shared between IB vs. SF and NB vs. SF comparisons. RT-qPCR was performed on eight selected genes (AALF001757, AAEL004052, AAEL004318, AALF008822, AALF010682, AALF011004, AALF011200, and AALF012673) to validate the results observed in the RNA-seq experiments (Figure 1C).

**FIGURE 2:**
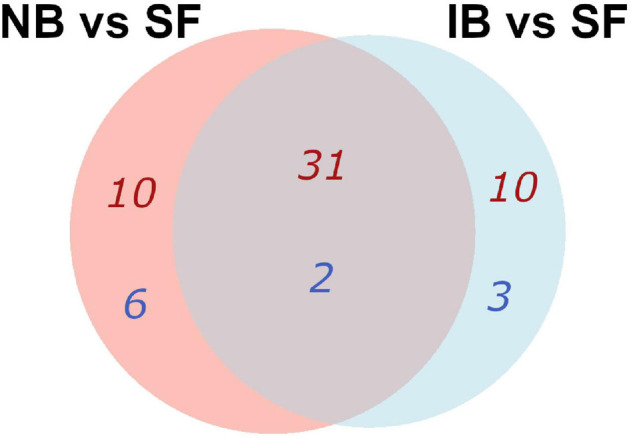
Venn diagram of KEGG pathways enrichment that were common and specific for the pairwise comparisons, using the differentially expressed genes (DEGs) orthologs of *Ae. aegypti* in the two comparison groups. The number of pathways up- in red, down- in blue.

Kyoto Encyclopedia of Genes and Genomes (KEGG) pathways enrichment analysis in the Search Tool for the Retrieval of Interacting Genes (STRING) platform was performed after the differentially expressed *Ae. albopictus* genes were assigned for the *Ae. aegypti* orthologs. Although the current annotation is relying upon the computational predictions, it was found that comparing IB vs. SF groups, forty-one upregulated and five downregulated metabolic pathways were enriched [False Discovery Rate (FDR) < 0.05]. Yet, in the NB vs. SF group, forty-one metabolic pathways were upregulated and eight downregulated (Supplementary Table 1) and thirty-one upregulated pathways were shared between both comparisons, involving into amino acid or lipid metabolism and oxidative stress; while two downregulated enriched pathways involving pentose and glucuronate interconversions, and nitrogen metabolism were common in both treatments (Figure 2). Furthermore, the IB group showed a significant decrease in expression of genes related to autophagy and the FoxO pathways. Meanwhile, the NB group showed significant changes in the metabolism of sphingolipids and pathways related to cytochrome P450 (Supplementary Table 1).

**FIGURE 3:**
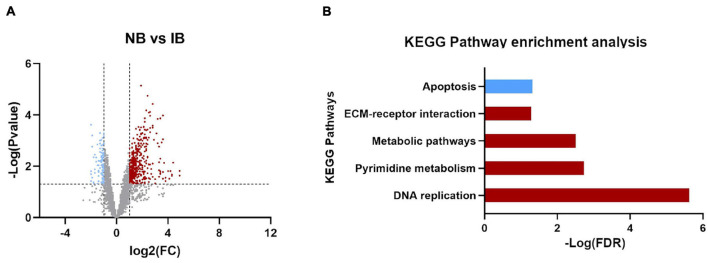
Differentially expressed genes influenced by the presence of active complement. **(A)** Volcano plot for the differentially expressed genes (DEGs), Normal Blood vs. Inactivated Blood. Upregulated (red plots) and downregulated (blue plots) DEGs. **(B)** KEGG pathways enrichment identified in DEGs between NB vs. IB. In red the upregulated pathways and in blue downregulated.

**FIGURE 4:**
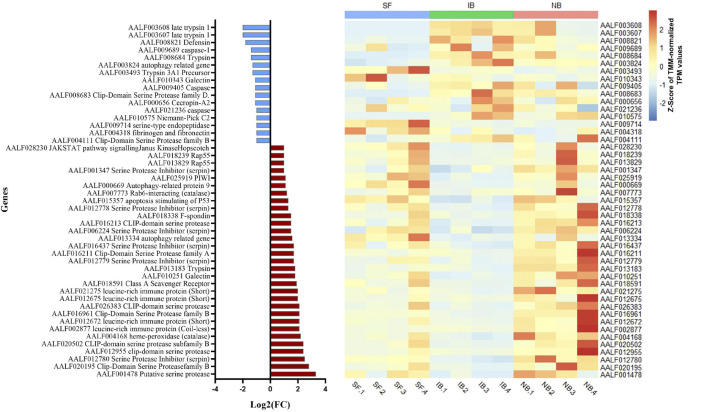
Fold change expression of immune related genes differentially expressed between NB and IB. Upregulated genes (red) and downregulated genes (blue).

Also, while identifying which genes showed significant differences between NB vs. IB to determine if heat inactivation of serum influences the expression of those genes, we observed 600 genes differentially expressed between these two groups, of which 481 (80.2%) were upregulated in the NB group (Figure 3A). Furthermore, the KEGG pathways analysis revealed four overexpressed and one downregulated metabolic pathways in the NB group (Figure 3B) one of these downregulated genes in the NB group is apoptosis while autophagy was found downregulated in the IB group. The DEG analysis showed forty-seven immune related genes, with thirty-one of those over-expressed in the NB group (Figure 4); twelve genes were related to lipid metabolism (eleven upregulated and one downregulated), ten genes were related to the cytochrome P450 family (five upregulated and five downregulated), while ten genes were associated with oogenesis (eighth upregulated and two downregulated).

### Gut Damage Detection

**FIGURE 5:**
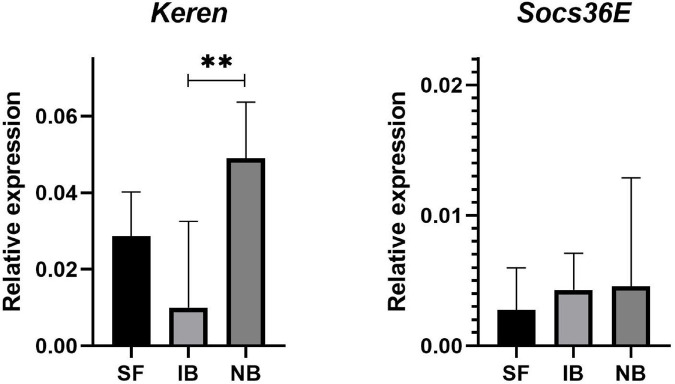
Relative expression of *Keren* and *Socs36E* compared to RP49 in midguts of *Ae. albopictus* mosquitoes sugar fed (SF), blood with heat-inactivated serum (IB), or normal serum (NB). ^∗∗^*P* < 0.01; ANOVA.

To determine if active complement protein was causing damage in mosquito guts, we evaluated the expression of two genes involved into molecular signaling pathways implicated in regeneration. The gene *Socs36E* is a target and negative regulator of the JAK/STAT pathway, and the *Keren* gene, which is one of the known epidermal growth factor receptor (EGFR) ligands, these ligands are highly expressed in the *Drosophila* midgut following stress-induce damaged. The qRT-PCR assays on dissected guts fed with the treatments showed that the *Socs36E* gene had no differences in its expression between the treatments, however, the *Keren* gene had a significant increase in the NB treatment compared to IB (Figure 5).

### Changes in the Bacteriome After Blood Meal

**FIGURE 6:**
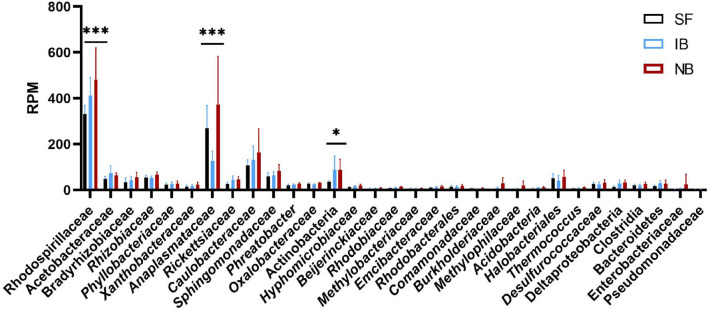
Composition of the *Ae. albopictus* bacteriome after feeding with sugar, inactivated blood, and normal blood. ^∗^*P* < 0.05, ^∗∗∗^*P* < 0.001; ANOVA.

**FIGURE 7:**
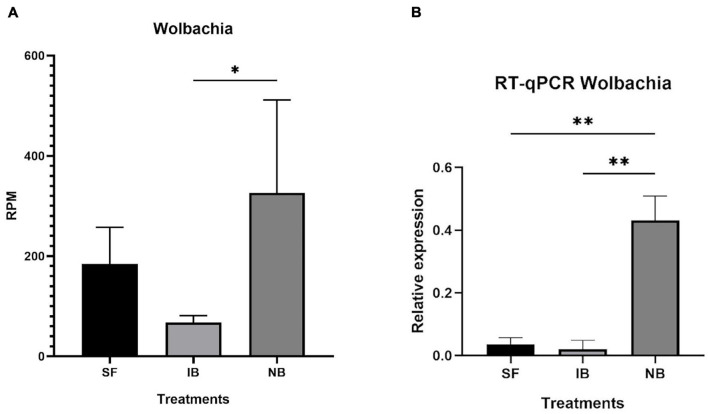
Relative abundance of *Wolbachia* in *Ae. albopictus* after each feeding treatment. **(A)** Relative abundance of *Wolbachia* reads normalize as reads per million reads mapped in the *Ae. albopictus* genome. **(B)** RT-qPCR of relative abundance of *Wolbachia* groEL transcript compared to *Ae. albopictus* RP49. ^∗^*P* < 0.05, ^∗∗^*P* < 0.01; ANOVA.

**FIGURE 8:**
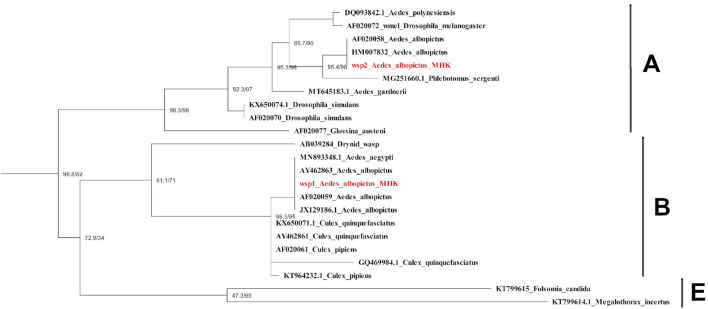
Phylogenetic tree of *Wolbachia* Wsp gene obtained from *Ae. albopictus* mosquitoes in Manhattan, KS, United States. Sequences identified in this study are labeled in red. Scale bar indicates the number of substitutions per site. Wsp: Wolbachia surface protein. KS: kansas.

After mapping the RNA-seq libraries to the *Ae. albopictus* genome, cleaning the presence of rRNA and assembling the remaining sequences to perform the microbiome identification, we found that 314,417 out of 314,422,593 (0.0001%) reads corresponded to bacteria. Then, we identified the presence of thirty-one operational taxonomic units (OTUs) (Supplementary Table 2). The number of sequences for each OTU was normalized as reads per million mapped reads (RPM). The values obtained were then compared between treatments to identify variations in the abundance of the different taxa sequences (Figure 6). The comparison test between all treatments showed significant differences in the number of sequences for Anaplasmatacea, Actinobacteria, and Rhodospirillaceae. Also, we observed that after 6 h post-blood feeding, Actinobacteria and Rhodospirillaceae showed an increase regardless the type of blood meal. However, further analysis of the Anaplasmatacea suggests a complex behavior, since in the IB group RPM decreased and the NB group presented a significant increase. Interestingly, the family Anaplasmataceae includes the genus *Wolbachia*, *Ehrlichia*, and *Neorickettsia*, so, we performed individual search to determine which of these families were the ones showing variation. The search revealed alterations in abundancy of *Wolbachia* reads abundance (Figure 7A). To validate the observed results, a PCR-based assay was performed to measure transcripts levels of the *Wolbachia* groEL gene, a single copy gene constitutively expressed (Chen et al., 2019). The relative levels of *Wolbachia* transcripts were normalized with respect to the *Ae. albopictus* Ribosomal protein 49 (RP49) gene expression (Figure 7B; Janeh et al., 2017). The detection of *Wolbachia* was in concordance with the transcripts results obtained from the abundance determination after taxonomic classification, confirming that the feeding the blood meal type influences the abundance of *Wolbachia*. Also, contigs mapped on the *Wolbachia* surface protein gene (Wsp) were used for a phylogenetic analysis using reference sequences of the Wsp gene from the different groups of this genus. The non-rooted tree obtained allowed us to determine the natural presence of *Wolbachia* from clade A and B in this population of *Ae. albopictus* studied (Figure 8).

### Virome Diversity and Stability After Blood Meal

**FIGURE 9:**
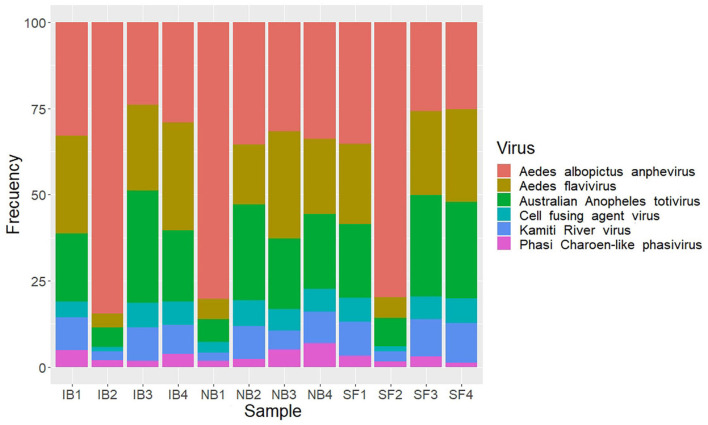
Stacked bar plots showing relative abundance of viral species identified after feed treatments.

Insect specific viruses (ISV) have been studied as potential modulators of immune responses in mosquitoes influencing vector competence (Öhlund et al., 2019). Previous studies have demonstrated that the presence of active serum factors influence pathogen populations in the midgut. Thus, we explored the possibility that the presence of active complement in mosquito midgut had any effect on ISV presence. To test this assumption, we used an RNA-seq approach to describe the RNA virome of *Ae. albopictus.* After removal of mosquito and ribosomal sequences, 103,384 contigs greater than 450 bp were obtained and were taxonomically assigned using DIAMOND and only eukaryotic viruses were taken into consideration. Eighty contigs were identified as viral OTUs, which were classified into six viral species belonging to four families (*Flaviviridae*, *Totiviridae*, *Xinmoviridae*, and *Phenuiviridae*) (Supplementary Table 2). The individual abundance of each viral species, measured as the number of reads mapped to each contig divided by the total amount of ribosomal RNA-depleted reads in the library and multiplied for 1,000,000 (i.e., reads per million, RPM), showed that the *Aedes albopictus* anphevirus (*Xinmoviridae*) was the most abundant virus in average (Figure 9). This virus was recently suggested and has been identified in RNA-seq samples from *Ae. albopictus* in multiple regions such as North America, Europe, and Asia (Manni and Zdobnov, 2020). The Australian Anopheles totivirus (*Totiviridae*) was the second most abundant in the samples evaluated. The most diverse family was Flaviviridae, in which we found the presence of Aedes flavivirus, Kamiti River virus, and Cell fusing agent virus. The Phasi Charoen-like phasivirus (*Phenuiviridae*) was the least abundant among the viral sequences found. A contrasting result with what is observed in *Ae. aegypti*, in which this virus superinfects it (Zhang et al., 2018). An overview of the metavirome visualized using Krona is shown in Supplementary Figure 1. The comparison among feeding treatments did not show significant changes in the abundance or the diversity of the virome (ANOVA *p*-value > 0.05), suggesting that blood feeding did not modify the viral community identified in the post-feeding time studied here (6 h).

## Discussion

*Aedes albopictus* mosquitoes are important vectors of arboviruses worldwide. Given the effect of blood meal and its active components on the mosquito transcriptome, we decided to evaluate the effect of complement on *Ae. albopictus* physiology and microbial composition. Specifically, this work is a continuation of our previous study in which we found that human complement proteins modify transcription in numerous *Ae. aegypt*i genes, affecting multiple metabolic pathways, including the immune system (Giraldo-Calderón et al., 2020). RNA-seq has served as a robust method to identify the gene expression profile of organisms and has been used to meta-taxonomy analysis (Cottier et al., 2018). *Ae. aegypti* mosquitoes, unlike *Ae. albopictus*, have a strong feeding preference for humans while *Ae. albopictus* shows an opportunistic feeding behavior on a wide range of hosts (from cold-blooded to warm-blooded animals) so we aim to study the effect of human complement factors in the later species (Richards et al., 2006; Delatte et al., 2010). This study provided insights into the effects of human complement protein activity over the gene expression, and the microbiome of *Ae. albopictus*.

We observed that human complement proteins influence the expression of at least 600 genes in *Ae. albopictus*. Because many *Ae. albopictus* genes have unknown function, full interpretation of the effect of human complement on gene expression could not be attained. Nevertheless, we found an alteration in the expression of 47 genes related to the immune system and may influence the pathogen survival ingested in the blood. One of the most interesting pathways found downregulated in the IB group was the autophagy pathway. This is a tightly regulated catabolic process whereby cells degrade intracellular components via the lysosomal machinery and it plays an important role in homeostasis maintenance, cell development, growth, and immunity (Chang and Neufeld, 2010). In humans, autophagy promotes cell survival under these stress conditions by recycling the organism own materials (proteins and organelles) (Kwon et al., 2019). This suggest that feeding with normal blood is important in maintaining the autophagy pathway functional. Interestingly, we observed that genes associated with apoptosis were downregulated in mosquitoes from the NB group. Gathering all this together suggest that the presence of active complement in human blood induces proliferation of gut cells and protects against apoptosis.

RNA interference is one of the most important anti-viral mechanism in mosquitoes (Blair, 2011). Several components of this pathway were found upregulated in the NB group. Specifically, we observed an increased transcript accumulation for Dicer 1 and PIWI genes. Dcr1 interacts with dsRNA-binding proteins (i.e., R3D1/Loqs) to process and load miRNA duplex into the RISC complex. Dcr1 displays some role in antiviral RNAi in mosquitoes through miRNA synthesis (Bernhardt et al., 2012), for example, an increase in miRNAs production in C6/36 cells has been detected in response to West Nile virus (Slonchak et al., 2014) and DENV infections (Yan et al., 2014). PIWI is a member of the PIWI small RNA (piRNA) pathway proposed to be involved in anti-viral defense (Marconcini et al., 2019), which has been identified in *Aedes* cultured cells and adult mosquitoes after infection by arthropod-borne alpha-, bunya-, and flaviviruses (Blair, 2019).

In our current study, we found the upregulated expression of ten genes of the CYP450 family suggesting that complement proteins contained in NB group may increase the expression of the oxidative stress response. Although, oxidative stress may damage host tissues if not controlled, it plays an important role in defense against pathogen replication and survival (Oliveira et al., 2011). In this regard, mechanisms protecting mosquito tissues from oxidative stress may have an impact on pathogen prevalence (Oliveira et al., 2017). Heme and iron contained in the blood meal may induce oxidative damage in the midgut (Whiten et al., 2017); as protection, hematophagous arthropods have evolved different mechanisms, including the synthesis of antioxidants such as catalase. In fact, a study showed that catalase levels increase at 24 and 36 h after a blood meal to recover to sugar fed mosquito levels once digestion is finished (Oliveira et al., 2017). Another study demonstrated that a blood meal decreases the production of reactive oxygen species through a heme-mediated activation of the protein kinase C (Oliveira et al., 2011).

Arboviruses are normally acquired through feeding and the midgut epithelial correspond to the first line of defense against infection (Franz et al., 2015). The midgut is a complex tissue, composed by several types of cells helping in the digestion and absorption of nutrients. In concordance with our observed upregulation of oxidative stress at 6 h post feeding, a recent study demonstrated that silencing of the *Ae. aegypti* factor erythroid-derived factor 2 (AeNrf2) increases reactive oxygen species levels and stimulates intestinal stem cell proliferation (Bottino-Rojas et al., 2018). The presence of mitotic cells in the midgut of *Ae. albopictus* (Janeh et al., 2017) and drastic changes in midgut cells have also been observed in *Culex* mosquitoes, which suggest that blood feeding induces midgut cells proliferation (Houk, 1977). The JAK/STAT and the EGFR pathways, and more specifically the *Socs36E* and *Keren* genes, respectively, are activated in *Ae. albopictus* midgut following feeding with human blood (Janeh et al., 2017). In our study, significant differences in the expression *Keren* were found when comparing midguts of mosquitoes fed with IB or NB, suggesting significant activation of the EGFR pathways regenerative signaling in midguts of mosquitoes fed with NB where the complement factors are still active. Although no significant differences among treatments were found in the expression of *Socs36E*, we found that the Hop ortholog in *Ae. albopictus* of a negative regulator of the JAK/STAT pathway in *Drosophila*, the JAK/STAT pathway signaling Janus Kinase Hopscotch (Hop) gene (AALF028230) associated with proliferation of diploid imaginal cells (Binari and Perrimon, 1994) was significantly upregulated in mosquitoes fed with NB, suggesting that both EGFR and JAK/STAT signaling pathways may be activated in NB mosquitoes with low suppression effect from *SOCS36E* (Baeg et al., 2005; Muller et al., 2005).

We found several genes involved in lipid metabolism and oogenesis upregulated in mosquitoes fed with NB. Although studying the specific blood components regulating oogenesis was out of the scope of this work, other studies suggest that lipid transport from the fat body to oocytes occurs within the first 30 h post blood feeding (Briegel et al., 2003) and that lipid storage and metabolism in the fat body after blood feeding are closely related to vitellogenesis (Pinch et al., 2021). Another study also suggests that blood feeding stimulates the release of two hormones, insulin-like peptide 3 and ovary ecdysteroidogenic hormone in *Ae. aegypti*, leading to the activation of the insulin/insulin growth factor signaling pathway in ovaries inducing oogenesis (Vogel et al., 2015). Taken all this information together, we believe that components present in non-heat inactivated serum have an effect on egg production signaling, but further research is needed to determine whether active human complement in the blood meal has a direct effect on oviposition or hatching rates.

On the other hand, multiple methods have been used to describe the host microbiome composition, like 16S and DNA-seq, and an accurate taxonomy classification based on RNA-seq reads can now be used to gather taxonomic information about the microbiota present in a biological sample without conducting additional sequencing, allowing efficiently and robustly identify bacteria, fungi, and viruses in the same sample (Cox et al., 2017). Herein, the meta-taxonomic analysis of the microbiome of *Ae. albopictus* showed the presence of at least 31 OTUs in the mosquito population analyzed. It has previously been known that the composition of *Ae. albopictus* microbiome varies between individuals, populations, even between field and colony mosquitoes (Seabournid et al., 2020); some studies have shown that 24–48 h after a blood meal, bacterial abundance increases, but diversity decreases (Wang et al., 2011; Terenius et al., 2012). In this study, we found that 6 h after blood-feeding the activity of Actinobacteria, Rhodospirillaceae, and Anaplasmataceae increase significantly. In beetles and hemipterans, Actinobacteria have been found as important nutrient processors aiding in the digestion of sugar and blood meals (Zucchi et al., 2012), and producing antibiotic barriers against pathogens (Seipke et al., 2011; Zucchi et al., 2011). Rhodospirillaceae is part of the phylum Proteobacteria, the dominant group in the *Ae. albopictus* microbiome (Juma et al., 2020). The results observed in this study suggest that the Rhodospirillaceae family may play a role in blood metabolism in mosquitoes. Interestingly, the increase in Anaplasmataceae was observed only in the NB treatment, suggesting that the observed change was caused by human complement proteins. We also found that, within Anaplasmataceae, the increase OTU corresponded to *Wolbachia*, a commonly found group in mosquitoes including *Ae. albopictus*. Interestingly, *Ae. albopictus* is naturally found super-infected with two *Wolbachia* strains, wAlbA and wAlbB (Sinkins et al., 1995; Zhou et al., 1998) as we found in this study. This genus of bacteria is a focus of interest because some strains have been shown to influence the vector competition of *Ae. aegypti* (Moreira et al., 2009). However, more studies are needed to elucidate the effect of blood meal on native strains of *Wolbachia* found in *Ae. albopictus* since previous studies suggest a minimal effect on the infection with human arboviruses (Mousson et al., 2012; Raquin et al., 2015; Joanne et al., 2017) in contrast to what is observed in the transinfected strains from *Drosophila melanogaster* used in *Ae. aegypti* (Fraser et al., 2017).

Our analysis revealed that the presence of the active complement in NB induced a significant increase in the number of *Wolbachia* sequences. Since *Wolbachia* is found in multiples mosquito tissues but is particularly abundant in the reproductive tissues of mosquitoes (Zouache et al., 2009; Joubert et al., 2016), we consider that changes in the *Ae. albopictus* transcriptome can serve as the triggering signal for the increase in *Wolbachia* activity. Possible signals linked to this could be an increase in the transcription of genes related to oogenesis and lipid metabolism. In this study we found an increase in the expression of genes related to lipid metabolism when the complement system is active. Mainly involving synthesis, transport, and storage of lipids. *Wolbachia* are unable to synthesize certain vital biomolecules, including lipids and amino acids, relying instead on the host metabolism for these compounds (Cho et al., 2011; Caragata et al., 2014) *in vitro* studies suggested a significant impact of *Wolbachia* replication and the depletion of lipids groups (sphingolipid, diacylglycerols, and phosphatidylcholines) that has been demonstrated increased in DENV-infected cells and that are required for optimal replication (Perera et al., 2012; Molloy et al., 2016). Consequently, further test with additional timepoints is necessary to determine if *Wolbachia* replication affects lipid metabolism upon feeding with active complement factors leading to changes in lipid metabolism or vice versa.

As we discussed before, previous studies have shown that the human complement system can remain active for up to 6 h post blood meal before its inactivation by blood digestion proteases (Margos et al., 2001; Simon et al., 2018; Pereira-Filho et al., 2020), and may interact with the mosquito cells and its associated gut microbiome. Also, since the presence of active human complement in mosquito midgut can decrease pathogen populations, we decided to investigate the impact of heat inactivation of human serum on the microbiome/virome of *Ae. albopictus* mosquitoes. To date, most of the conducted mosquito microbiota studies have focused on the bacterial component in mosquitoes and their possible intrinsic roles in mosquito biology. However, numerous insect-specific viruses (ISV) have been reported in culicids, and increasing evidence suggest that ISVs might influence the mosquito physiology as well as its ability to transmit important arboviruses (Öhlund et al., 2019). Some studies, mainly with *Ae. albopictus* from Asia, have begun to reveal information about the existence of numerous viruses that remain stable in mosquito populations, apparently transmitted vertically (Zhao et al., 2019; Kubacki et al., 2020; Shi et al., 2020). For instance, a study reports that although *Ae. albopictus* cell lines are permissive to the Phasi Charoen-like virus, it was only found in *Ae. aegypti* cells. Yet the presence of such virus interrupts dengue and Zika virus replication during co-infection with Cell-fusing agent virus (Schultz et al., 2018). Still, more studies are needed to elucidate how ingestion of blood can affect the structure of the viral community in mosquitoes and whether these communities can influence their ability to prevent or facilitate pathogen colonization.

Through RNA sequencing it has been possible to know the presence of numerous viruses in mosquitoes, however, obtaining this information is limited by the sequencing technology and depth of sequencing, for which some methods have been suggested to carry out viral enrichment of libraries (Sadeghi et al., 2018; Shi et al., 2020). Diversity of insect viruses found in this study was low, compared to previous studies, in which they have proposed the presence between 1 and 23 viral species in *Ae. albopictus* populations, however, many of them unclassified (Kubacki et al., 2020; Shi et al., 2020). The number of viruses identified in this work could be affected by the astringent identification criteria (contigs identity and cover >60%), sequencing depth, and having used F1 colonized mosquitoes. In this regard, it is possible that the conditions used to raise the F1 in the laboratory, such as the use of distilled water and larvae food, influenced the microbial composition (Martinson and Strand, 2021). A recent study compared the virome of *Ae. albopictus* derived from field and from colonies and concluded that both in populations, the virome seem to be stable through the life cycle with each group of mosquitoes displayed the respective “core virome” (Shi et al., 2020). Still, it has been reported that the virome diversity in lab *Ae. albopictus* is lower than in field mosquitoes, probably due to the less complex environment and the availability of clean water and food resources (Shi et al., 2020). In this study, we found the presence of six viruses associated with insects that are present in this population of *Ae. albopictus* studied, which were not affected after the blood meals. Therefore, we consider that this set of viruses found is a representation of the natural core virome of *Ae. albopictus*, which seems to be mainly made up of flaviviruses, and is kept in circulation through vertical transmission (Shi et al., 2020). The *Ae. albopictus* anphevirus was the virus with the highest number of average sequences, however, in most samples it presented a proportion like Aedes flavivirus and Australian Anopheles totivirus, therefore, its apparent dominance could be caused as an artifact of the sequencing of a batch of samples. The effect of anphevirus on the biology of *Ae. albopictus* mosquitoes is largely unknown, however, it has been reported that another Anphevirus (Aedes Anphevirus) has complex interplays with *Wolbachia* and can interfere with DENV replication (Parry and Asgari, 2018). In addition, an *Ae. albopictus* anphevirus has been reported as globally distributed and may interfere with vector competence through modulation of the RNAi pathway (Manni and Zdobnov, 2020).

**FIGURE 10:**
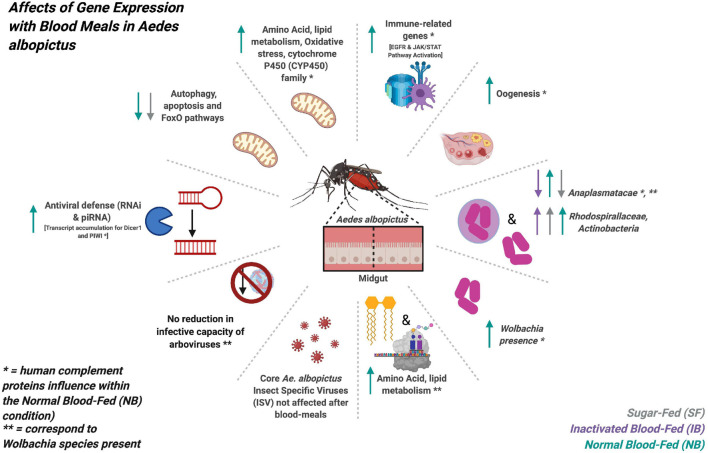
Graphic summary of the main findings obtained in the transcriptome expression and microbiome changes of *Ae. albopictus* after feeding with sugar, heat-inactivated, and normal blood.

In conclusion, our results show that after a blood meal, human complement proteins influence the transcription of multiple *Ae. albopictus* genes, modifying functions such as the immune system, lipid metabolism, oxidative stress, and oogenesis. Also, the presence of *Wolbachia* increases after the ingestion of a normal blood meal, which suggests a direct or indirect effect of human complement in the activity of these bacteria (a figure summarizing the main findings of this study can be found on Figure 10). Therefore, it will be necessary to consider the characteristics of the blood meal as a factor that can influence vector competence in mosquito infection studies. This study evaluates the changes that occur 6 h post-feeding, however, physiological changes can manifest up to 36 h, therefore, future studies should evaluate different post-feeding times to fully identify the derived changes in gene expression, the microbiome and the virome, caused by the blood feeding, and specially by human complement proteins.

## Materials and Methods

### Mosquitoes Sampling and Maintenance

Mosquitoes were collected using ovitraps set in Manhattan, KS, United States. Each trap consisted of a 10 cm diameter black plastic cup, a rectangle (8 cm × 15 cm) of 3MM CHR filter paper (Whatman, Maidstone, United Kingdom), and 350 ml of rainwater. Six traps were set weekly from June 10th to August 26th, 2019, and from June 2nd to July 28th, 2020. All collections were made in urban settings. The traps were left in the field for 7 days and then transported to the lab. Filter papers were checked under a microscope for the presence of *Aedes*-like eggs, and the content of each trap was emptied into a transparent tray and checked for the presence of mosquito larvae.

Mosquito eggs were allowed to hatch in the breeding site water. F0 larvae and pupae were maintained in distilled water with dog chow as a food source. Mosquitoes were reared at 27 ± 1°C, 75 ± 5% RH, with a photoperiod of 16:8 h (L:D). Adults were maintained on 10% sucrose *ad libitum*. Randomly picked fourth-instar larvae were selected for morphological identification, and newly emerged adults were identified by visual inspection. F0 adults were allowed to mate for 2–3 days following emergence, then were offered blood meals twice a week, and allowed to lay eggs. Mosquitoes were fed for 60 min, at room temperature, using a Hemotek feeder system warmed at 37°C and 1 ml of bovine blood (Lampire Biological Laboratories, Pipersville, PA, United States). A 50 ml plastic cup with distilled water and a 42.5 mm circle of Whatman Grade 1 filter paper was placed inside F0-adult cages to collect F1 eggs. All F1 eggs were pooled and hatched in distilled water. F1 Female mosquitoes (*n* = 20 per experiment) 5–10 days-old were feed with a mixture of human red blood cells in a 1:1 ratio with either heat-inactivated or normal non-inactivated autologous plasma.

### Blood Source

The protocols included in this study were approved by the Kansas State University Review Board (IBC# 1205, IRB #8811). Human peripheral blood was collected in tubes containing ethylenediaminetetraacetic acid (EDTA), then, plasma was separated from the red blood cells (RBCs) by centrifugation. Red blood cells (RBCs) were washed three times in 1X PBS and kept at 4°C until use. Plasma was separated in two categories, normal non-inactivated and heat-inactivated 30 min at 56°C. Aliquots were stored at −20°C until use.

### Mosquito Blood-Feeding

Female adult mosquitoes were fed with either heat-inactivated blood plasma (500 μL inactivated plasma + 500 μL of homologous packed RBC), or normal non-inactivated plasma (500 μL normal plasma + 500 μL homologous packed RBC). Mosquitoes were fed during 30 min at room temperature using 1 mL of blood mixture in a Hemotek feeder maintained at 37°C. Blood-fed females were placed in a separate cage. Six hours post-feeding, whole abdomens or midguts were dissected and transferred to 1.5-mL tubes with 200 μL of DNA/RNA shield (Zymo Research, Irvine, CA, United States), in pools of five abdomens or midguts. Tissue of females fed sugar solution 10% were used as control. Mosquito tissues was homogenized in ZR Bashing-Bead lysis tubes.

### Mosquito RNA Isolation and Sequencing

Mosquito total RNA was extracted from the homogenizated per the manufacturer’s instructions by Quick-RNA miniprep kit (Zymo Research, Irvine, CA, United States). Each treatment was conducted in four replicates (*n* = 5 mosquitoes per replicate). The Ribo-Zero Gold Kit (Human/Mouse/Rat) was used to deplete ribosomal RNA (New England Biolabs, Ipswich, MA, United States). A total of 100 ng of depleted RNA per sample was sent for sequencing to The Genome Sequencing Laboratory at The University of Kansas (Lawrence, KS, United States), where the sample QC, libraries preparation, and were sequenced as 150 pb single reads in the Illumina HiSeq [NextSeq Mid-Output (MO)].

### Transcriptome Analysis

Raw files were processed for removal of Illumina adaptor sequences, trimmed and quality-based filtered using Fastp software v.0.20.0 (Chen et al., 2018). The remaining high-quality reads were mapped onto the reference genome of *Ae. albopictus* assembly AaloF1.2 using STAR v2.7 (Dobin et al., 2013). The unmapped reads were separated to perform the microbiome analysis. Mapped reads were counted using RNA-Seq by Expectation-Maximization (RSEM) v1.3.3 (Li and Dewey, 2011). The expression of genes in different libraries was normalized by the Trimmed Mean of *M*-values (TMM), and differential expressed genes (DEG) (Log2FC ≥ | 1| and Benjamini–Hochberg adjusted *p*-value ≤ 0.05) were identified using edgeR v3.32.1 (Robinson et al., 2010), which assumes a Negative Binomial Distribution model.

The gene description, gene ontology (GO) terms and orthologs of *Ae. aegypti* of DEG were obtained using the BiomaRt package v.2.44.1 (Durinck et al., 2005). To identify DEG-enriched metabolic pathways, a Kyoto Encyclopedia of Genes and Genomes (KEGG) pathways enrichment analysis was performed in the Search Tool for the Retrieval of Interacting Genes (STRING) v.11.0 platform^1^, using the *Ae. aegypti* orthologs.

### Verification of DEG and Gut Damage Detection by RT-qPCR

To confirm the results of the RNA-seq analyses, relative expression levels of eight genes (Supplementary Table 3) differentially expressed between sugar fed and blood fed mosquitoes were chosen. For real-time quantitative PCR analysis a total of 100 ng of RNA were used for cDNA synthesis with the High-Capacity cDNA Reverse Transcription Kit (Applied Biosystems) according to manufacture instructions. Real-time quantitative PCR reactions of 10 μl were performed in triplicate with SYBR Green Supermix (Bio-Rad). Real-time quantitative PCR reactions were run on a real Applied Biosystems QuantStudio 3. No primer dimer was detected when inspecting the melting curves.

Fold-changes in gene expression between IB and NB mosquitoes were derived by the 2(-Delta Delta C(T)) method (Livak and Schmittgen, 2001), using the constitutive ribosomal protein 49 (RP49) as housekeeping gene, and sugar fed treatment as control. Samples were considered negative if the cycle threshold (Ct) value was greater than 30.

For the detection of cell damage, RNA from dissected guts. Four biological replicates were used for each treatment, each one with three dissected midguts. The RT-qPCR assays were performed as described elsewhere (Janeh et al., 2017). Ct values for target genes were normalized to Rp49 and compared to controls using the delta Ct method.

### Virome and Bacteriome Analysis

The unmapped sequences in the genome of *Ae. albopictus* were used to identify the set of viruses and bacteria present. First, a custom database of ribosomal RNA sequences was generated using SILVA v132 LSU, SSU and 5S rRNA (RF00001), 5.8S rRNA (RF00002), tRNA (RF00005), Ribonuclease P (RF00010, RF00011, and RF00373). And the software SortMeRNA v.2.1 (Kopylova et al., 2012) was used to identify ribosomal sequences in the transcriptome data using an *e*-value cut-off of 10^–5^ (Zhao et al., 2019). Reads with an identity >60% and 60% of the read length covered were marked as ribosomal and excluded from further analysis.

The remaining sequences were assembled using SPAdes v.3.12.0 (Bankevich et al., 2012), and contings greater than 450 nt were classified using DIAMOND v.2.0.8 (Buchfink et al., 2014) against the nr viral and bacterial databases (updated February 2021) using the sensitive mode for taxonomic annotation with an *e*-value cut-off of 10^–5^ and bit score cut-off of 100 (Zhao et al., 2019; Kubacki et al., 2020). KronaTools v.2.7.1 (Ondov et al., 2011) were used to parse the output file of DIAMOND, which found the least common ancestor of the best 25 DIAMOND hits (based on BLASTx score). The number of reads for each contig present in the detected taxonomic units was normalized using the RPM method with respect to the reads mapped in the *Ae. albopictus* genome per library and GraphPad Prism software v.9.0.1 was used to determine modifications in viral and bacterial abundance.

### *Wolbachia* RT-qPCR Detection

We used an RT-qPCR to verify the presence of *Wolbachia*. Specific primers were designed for the groEL gene (Supplementary Table 3), which is a constitutive gene. RT-qPCR was performed in the same way as described above. The relative abundance of *Wolbachia* was estimated with respect to the mosquito housekeeping RP49 expression.

### Phylogenetic Analysis

**FIGURE 11:**
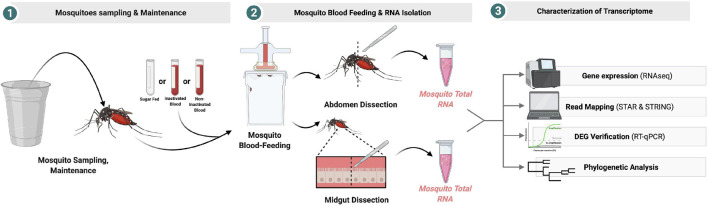
Graphic summary of the experimental design.

The contigs mapped on the *Wolbachia* surface protein (Wsp) gene were extracted and aligned with respect to 21 reference sequences. Alignments of the sequences were performed with MAFFT v.7.475 (Katoh and Standley, 2013) using the most accurate algorithm, L-INS-I, with 1,000 cycles of iterative refinement. The phylogenetic tree was reconstructed from 1,000 ultrafast bootstrap ML tree replicates using IQ-TREE v1.6.12 (Nguyen et al., 2015) with best-fit model selection by ModelFinder (Kalyaanamoorthy et al., 2017). FigTree v.1.4.4^2^ was used for phylogenetic tree visualization. A figure summarizing the methods used and the main finding of this study can be found on Figure 11.

## Data Availability Statement

The data presented in the study are deposited in the SRA repository, accession number PRJNA722323. The data are publicly available at https://www.ncbi.nlm.nih.gov/bioproject/PRJNA722323.

## Ethics Statement

The studies involving human participants were reviewed and approved by the Kansas State University (IRB#8811). The patients/participants provided their written informed consent to participate in this study.

## Author Contributions

BL-R: conceptualization and funding acquisition. BL-R, PR-L, SF, and AC-T: methodology. BL-R, AC-T, PR-L, AH-R, CM, MR-B, and JH: experiments. AC-T: bioinformatics analysis and original draft preparation. BL-R, AC-T, YP, and GR-U: writing – review and editing. All authors contributed to the article and approved the submitted version.

## Conflict of Interest

The authors declare that the research was conducted in the absence of any commercial or financial relationships that could be construed as a potential conflict of interest.

## Publisher’s Note

All claims expressed in this article are solely those of the authors and do not necessarily represent those of their affiliated organizations, or those of the publisher, the editors and the reviewers. Any product that may be evaluated in this article, or claim that may be made by its manufacturer, is not guaranteed or endorsed by the publisher.

## Footnotes

1
https://string-db.org/

2
http://tree.bio.ed.ac.uk/software/figtree/

## Supplementary Material

Supplementary Figure 1 Virome classification of *Ae. albopictus* samples using DIAMOND and the lowest common ancestor (LCA) approach, with results visualized by Krona. Normalized abundance of viral reads aligning to the indicated virus species.

Supplementary Table 1 KEGG metabolic pathways differentially expressed between the feeding treatments in *Aedes albopictus*, using the orthologs of *Aedes aegypti.*

Supplementary Table 2 Bacteriome and virome detected in *Aedes albopictus*. Number of contigs and reads per OUT, identity percentage, and Average of Log (*e*-value).

Supplementary Table 3 List of primers used in the RT-qPCR assays.
