# Supplementary figures and images for: Blood Meals With Active and Heat-Inactivated Serum Modifies the Gene Expression and Microbiome of Aedes albopictus
Source: Front Microbiol. 2021 Sep 9;12:724345. doi: 10.3389/fmicb.2021.724345 (PMC8458951; doi:10.3389/fmicb.2021.724345)

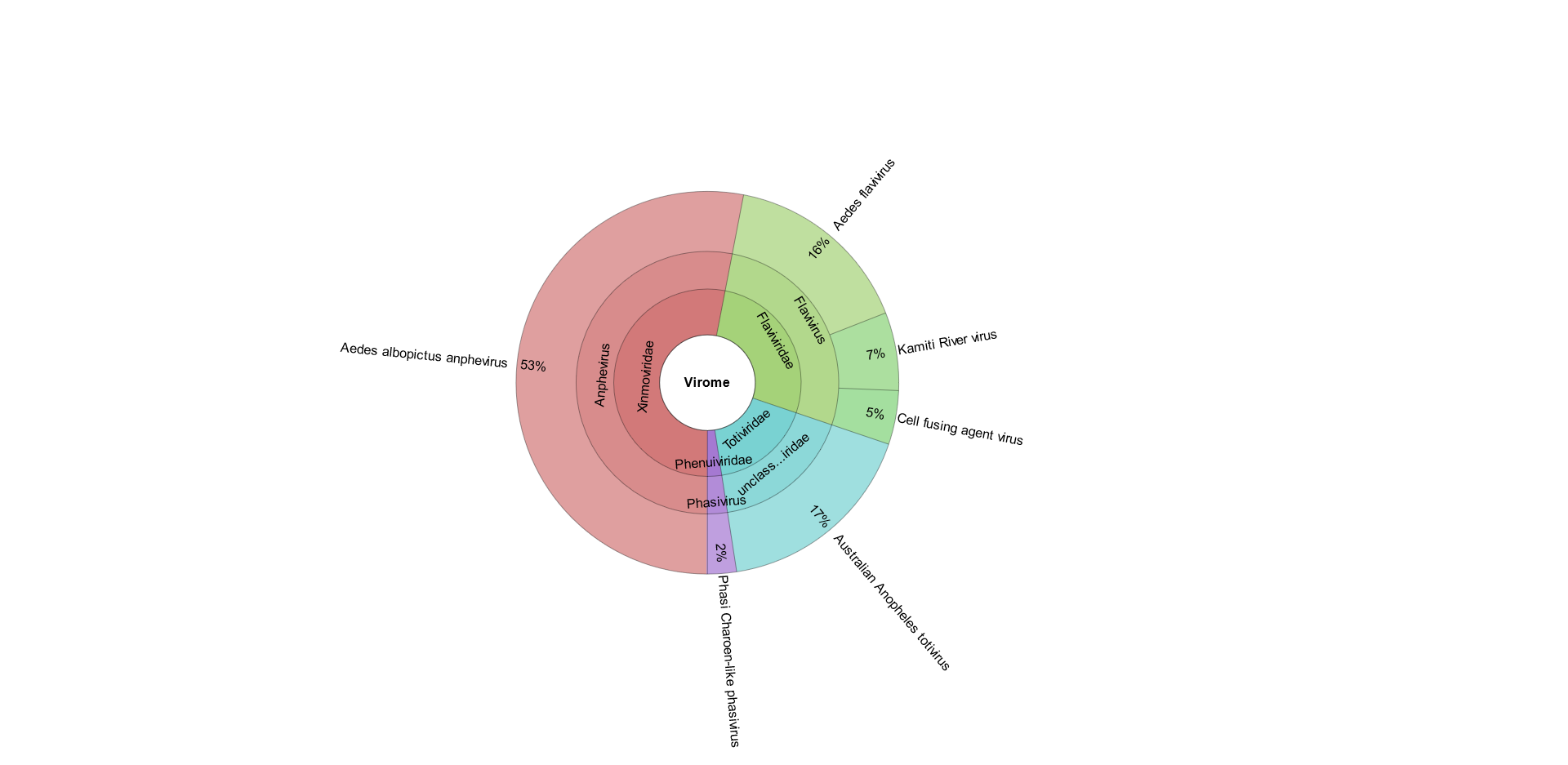

Supplement: Supplementary Figure 1 — Virome classification of Ae. albopictus samples using DIAMOND and the lowest common ancestor (LCA) approach, with results visualized by Krona. Normalized abundance of viral reads aligning to the indicated virus species. [file Data_Sheet_1.zip › Image 1.PNG]
